# Detection of prions in the urine of patients affected by sporadic Creutzfeldt–Jakob disease

**DOI:** 10.1002/acn3.51919

**Published:** 2023-10-10

**Authors:** Sandra Pritzkow, Frank Ramirez, Adam Lyon, Paul E. Schulz, Brian Appleby, Fabio Moda, Santiago Ramirez, Silvio Notari, Pierluigi Gambetti, Claudio Soto

**Affiliations:** ^1^ Department of Neurology, Mitchell Center for Alzheimer's Disease and Related Brain Disorders University of Texas McGovern Medical School at Houston Houston Texas USA; ^2^ Department of Pathology Case Western Reserve University Cleveland Ohio USA; ^3^ Division of Neurology 5 – Neuropathology Fondazione IRCCS Istituto Neurologico Carlo Besta Milan Italy

## Abstract

**Objective:**

Currently, it is unknown whether infectious prions are present in peripheral tissues and biological fluids of patients affected by sporadic Creutzfeldt–Jakob disease (sCJD), the most common prion disorder in humans. This represents a potential risk for inter‐individual prion infection. The main goal of this study was to evaluate the presence of prions in urine of patients suffering from the major subtypes of sCJD.

**Methods:**

Urine samples from sCJD patients spanning the six major subtypes were tested. As controls, we used urine samples from people affected by other neurological or neurodegenerative diseases as well as healthy controls. These samples were analyzed blinded. The presence of prions was detected by a modified version of the PMCA technology, specifically optimized for high sensitive detection of sCJD prions.

**Results:**

The PMCA assay was first optimized to detect low quantities of prions in diluted brain homogenates from patients affected by all subtypes of sCJD spiked into healthy urine. Twenty‐nine of the 81 patients affected by sCJD analyzed in this study were positive by PMCA testing, whereas none of the 160 controls showed any signal. These results indicate a 36% sensitivity and 100% specificity. The subtypes with the highest positivity rate were VV1 and VV2, which combined account for about 15–20% of all sCJD cases, and no detection was observed in MV1 and MM2.

**Interpretation:**

Our findings indicate that potentially infectious prions are secreted in urine of some sCJD patients, suggesting a possible risk for inter‐individual transmission. Prion detection in urine might be used as a noninvasive preliminary screening test to detect sCJD.

## Introduction

Prion diseases are a group of fatal and infectious neurodegenerative disorders affecting humans and various species of animals.^1^ Sporadic Creutzfeldt–Jakob disease (sCJD) is the most common prion disease in humans with an average incidence of one to two individuals per million people each year worldwide. The disease is transmitted by an unconventional infectious agent termed prion, which is composed exclusively of the misfolded prion protein (PrP^Sc^).^1^ Close to 500 cases of CJD have been transmitted iatrogenically between humans by the use of biological products from human origin or by medical procedures including organ transplant and blood transfusion.^2^ In addition, >200 cases of variant CJD (vCJD) were caused by exposure to cattle prion‐contaminated material.^3^

Despite impressive advances in the knowledge about the molecular and cellular basis of CJD, the disease remains incurable and without any treatment option to offer to patients. One of largest problems to develop an efficient treatment is that when prion diseases manifest clinically, a substantial part of the brain is severely and irreversibly damaged, making very difficult to produce a therapeutic benefit.^4^ It is likely that the best option would be to identify people on the way to develop CJD before they develop clinical symptoms and substantial damage in the brain. For this purpose, it would be necessary to have a noninvasive and inexpensive diagnostic test that can be applied to healthy individuals at risk. Combining a noninvasive, early diagnosis with a safe disease‐modifying therapy will offer the best possibility for treatment.

Although prion diseases mostly affect the brain, it is well‐established that infectious prions can be detected in many peripheral tissues and biological fluids.^5^ The relative amount of prions in the brain and the periphery varies substantially depending on the specific disease. For example, it is well‐known that PrP^Sc^ is distributed all over the body in cases of vCJD, whereas is mostly restricted to the brain in sCJD.^6^ Indeed, we and others detected PrP^Sc^ with sensitivity and specificity approaching 100% in blood and urine samples of vCJD patients.^7^, ^8^, ^9^, ^10^ Contrary to the situation in vCJD, despite few reports suggesting that prions are present in blood and urine of sCJD patients,^11^, ^12^ the limited number of studies makes still unclear how general is the presence of sCJD prions in blood and urine. This may represent a potential risk for prion transmission and a source for diagnostic test development.

An additional issue adding to the complexity of human prion diseases is that sCJD actually consists of several clinically and pathologically distinct conditions commonly referred to as sCJD subtypes.^13^ At the molecular level, the sCJD subtypes hinge on (i) the methionine/valine (MV) polymorphism at codon 129 generating three PrP genotypes (MM, MV, VV) and (ii) the consistent association with two conformationally distinct PrP^Sc^ variants referred to as types 1 and 2.^14^, ^15^ Accordingly, sCJD subtypes are identified as MM1, MM2, MV1, MV2, VV1, and VV2. The MM2 and MV2 subtypes can be separated in two groups according to clinicopathological differences, referred as MM2C, MM2T, MV2C, and MV2K.

Here, we used an ultrasensitive test for prion detection, termed protein misfolding cyclic amplification (PMCA),^4^, ^16^ to study whether PrP^Sc^ is detectable in urine samples from patients affected by six common subtypes of sCJD.

## Materials and Methods

### Patient samples

Urine samples from patients and controls were collected at three different Research Centers: The National Prion Disease Pathology Surveillance Center at Case Western Reserve University (Dr. Appleby, Notari and Gambetti), Fondazione IRCCS Istituto Neurologico Carlo Besta, Division of Neurology 5—Neuropathology, Milan, Italy (Dr. Moda) and the University of Texas McGovern Medical School at Houston (Dr. Schulz). Urine was noninvasively collected in 10–40 ml volumes from 81 patients affected by sCJD with different subtypes as classified by Gambetti and coworkers.^13^ In total, 24 MM1, 16 MM2, 8 MV1, 11 MV2, 3 VV1, and 19 VV2 patients were studied. Patients presented clear symptoms and disease progression of sCJD, and definitive diagnosis was achieved postmortem by conventional biochemical and neuropathological assessment. As controls, we tested 66 samples from healthy individuals and 94 patients affected by a variety of confirmed other neurodegenerative (Alzheimer's, Parkinson's disease, frontotemporal dementia, cerebellar ataxia, multiple system atrophy, progressive supranuclear palsy, traumatic brain injury, mild cognitive impairment, motor neuron disease) or neurological diseases (brain tumor, multiple sclerosis, epilepsy, neuropathy, intracranial hypertension, hydrocephalus, vasculitis, extrapyramidal syndrome, migraine, charcot marie tooth syndrome, brain aneurism, myasthenia gravis, vascular encephalopathy, spinal myoclonus, stroke). Sample collection was done with informed consent and was approved by the Institutional Review Boards of each institution.

### Sample processing

Urine samples were processed by iron oxide magnetic extraction (IOME). Aliquots of 500 μL from patient urine were incubated in the presence of 0.5 mg/mL of iron oxide nanoparticles for 2 h with inversion at room temperature, following a procedure previously reported by Hoover and colleagues.^17^ Thereafter, a magnet was used to draw the iron oxide to the bottom of the tube, and the supernatant was removed. The makeshift iron oxide pellet was resuspended in 10% brain homogenate from both TgHuPrP^C^ 129M or 129V transgenic mice, supplemented with 6 mM EDTA, 0.05% digitonin, 0.01% sodium tripolyphosphate, and 100 μg/mL heparin.

### Prion detection by PMCA

PMCA was conducted as previously described,^10^, ^18^ with the modifications indicated below. For the preparation of 10% brain homogenate, brains were harvested from transgenic mice expressing Hu129M or Hu129V PrP^C^, and manually homogenized on ice, diluted in conversion buffer (1X phosphate‐buffer saline, 150 mM sodium chloride, 1% Triton X‐100) with the addition of protease inhibitors (Roche, complete EDTA‐free). To remove large cellular debris, 10% brain homogenate was clarified by centrifugation (810 x g) for 1 min at constant 4°C. For high amplification of sCJD prions, the 10% brain homogenate was treated with various supplemental reagents (6 mM EDTA, 0.05% digitonin, 0.01% sodium tripolyphosphate, and 100 μg/mL heparin). The mixture was subjected to three rounds of PMCA consisting of cycles of 29 min incubation followed by 1 min sonication, as described.^10^, ^18^ Upon completion of PMCA, PrP^Sc^ signal was detected by western blot using the anti‐prion specific primary antibody 6D11, after initially conducting proteinase K (PK) digestion (100 μg/μL, 1 hour, 37°C, 650 rpm) and running SDS‐PAGE. Samples were done by duplicate and were considered positive if at least one of the replicates showed PrP^Sc^ signal in the third round of PMCA.

### Statistical analysis

To analyze whether detectability by PMCA was statistically significantly different in distinct sCJD subtypes, we employed the Fisher's exact test, using GraphPad Prism version 7. The Fisher's exact test was employed because the number of samples is small, and the values are binary (positive and negative) and categorical. We compared whether each sCJD subtype produced a different result from the average of the entire group minus that subtype. Differences were considered significant with *p* < 0.05.

## Results

**Figure 1:**
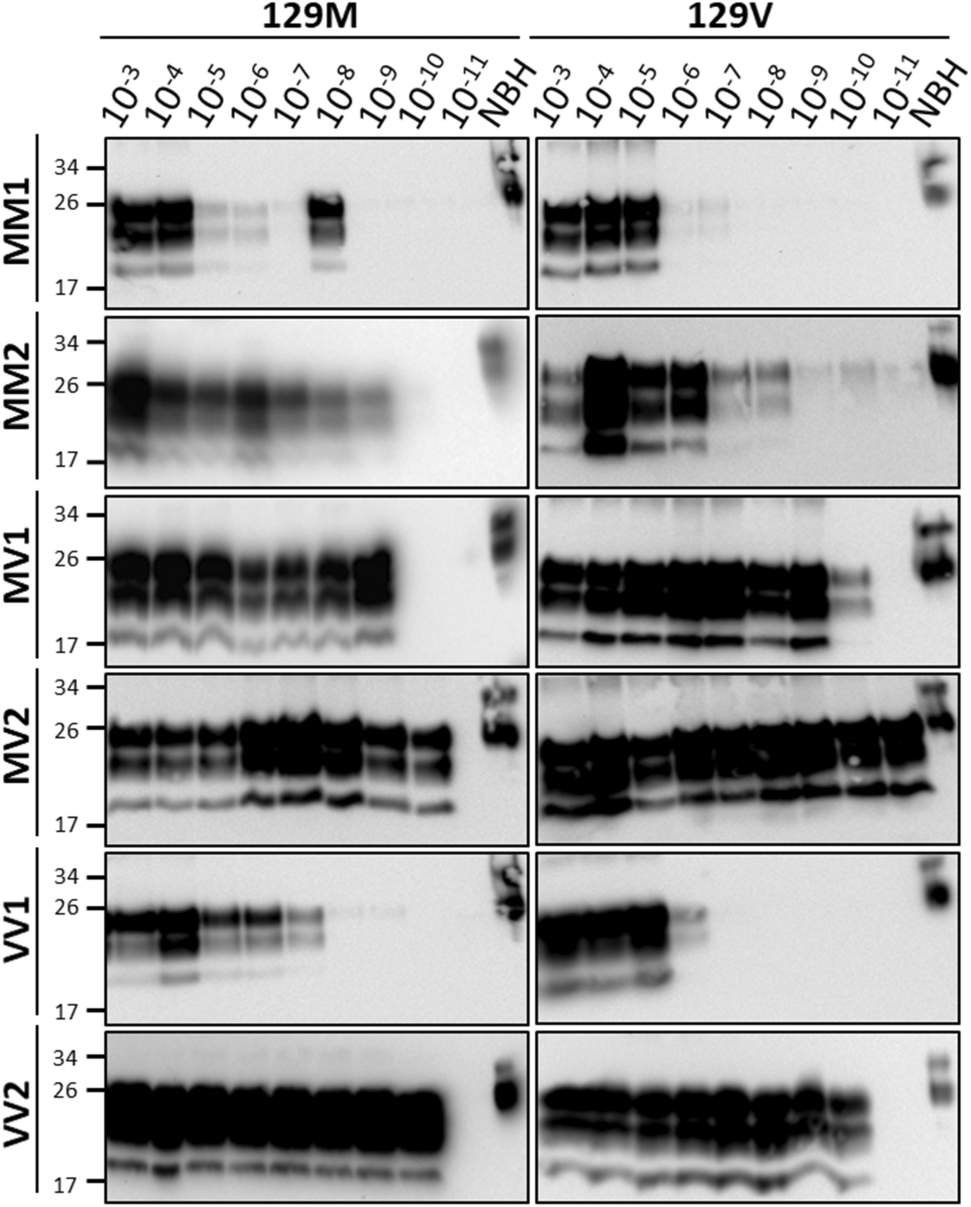
Optimization of PMCA for each subtype of sCJD. To evaluate baseline max amplification of each sCJD subtype, serial dilutions of sCJD brain homogenate (BH) were added to 10% w/v BH from transgenic mice overexpressing HuPrP^C^ with either methione or valine at position 129. Three rounds of PMCA were conducted consisting of 144/96/96 PMCA cycles; each PMCA cycle consists of a 20 sec sonication pulse every 29 min and 40 sec of incubation. PMCA amplification was visualized with western blotting after proteinase K treatment (100 μg/μL for 1 h) and probed with the prion protein specific antibody, 6D11 (1:30,000 dilution). The figure shows the results of the third round of PMCA for each sCJD subtype using either 129M or 129V transgenic mice BH as substrate. Normal brain homogenate (NBH) from humanized transgenic mice without PK digestion was loaded as a migration control. Numbers in the left show the position of molecular weight standards.

**Figure 2:**
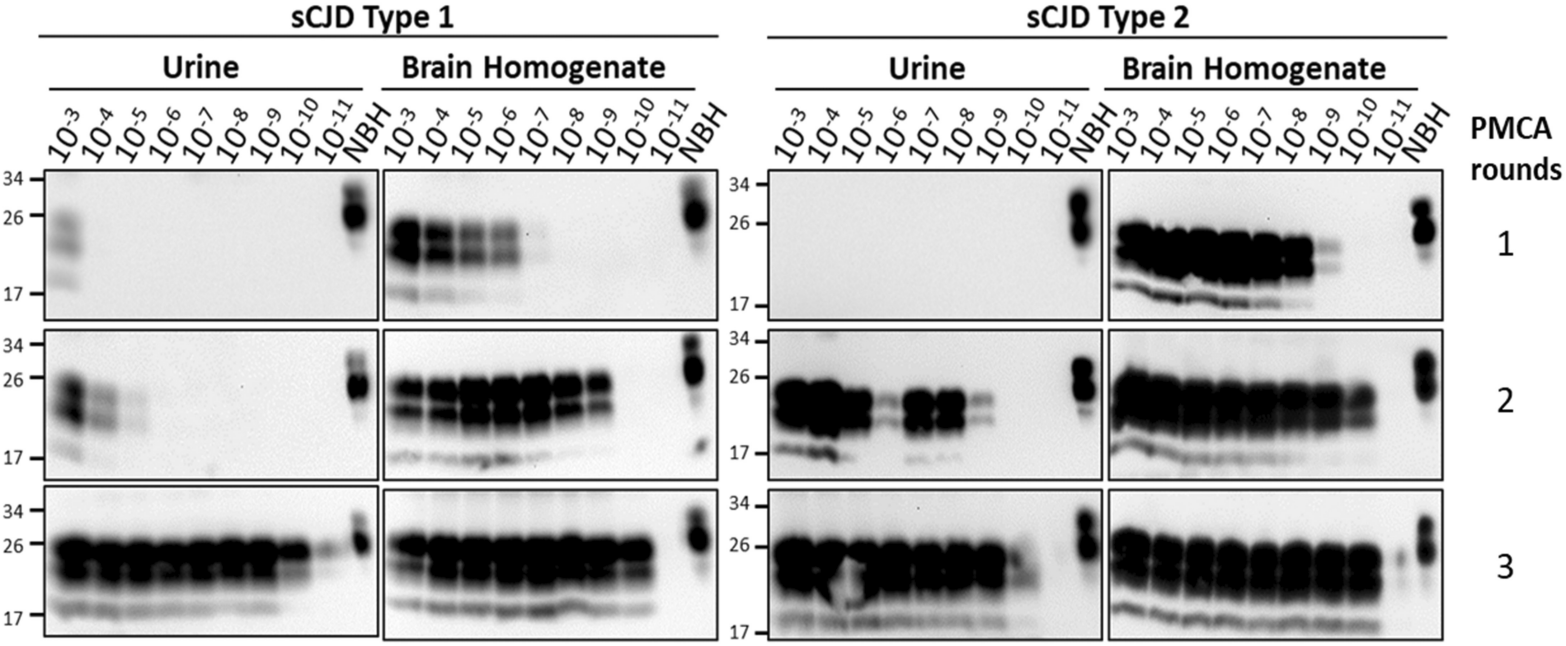
Effect of urine on PMCA efficiency. In order to evaluate the PMCA efficiency in the context of urine, different dilutions of sCJD type‐1 (MV1) or type‐2 (VV2) BH were spiked into healthy urine and processed via iron oxide magnetic extraction (IOME). Three serial rounds of PMCA were done, and the PrP^Sc^ product was detected by western blot after PK digestion. As before, normal brain homogenate (NBH) without PK digestion was loaded as a migration control. Numbers in the left show the position of molecular weight standards.

Detection of sCJD prions by PMCA has usually yielded a rather low rate of amplification, contrasting the ultrasensitive detection of vCJD by this technique.^4^, ^5^ Thus, first we tested several modifications of the PMCA procedure to increase amplification efficiency. These studies were done with serial dilutions of brain homogenate from patients affected by each of the different sCJD subtypes, defined as previously described based on the genotype at position 129 of the prion protein (M or V) and the electrophoretic mobility of PrP^Sc^ after PK digestion (type 1 or 2). As reported by Parchi, Gambetti and colleagues,^13^, ^15^ this classification correlates with differences in clinical and/or neuropathological presentation of the disease. After testing many changes, we achieved a protocol for high sensitive detection of all major subtypes of sCJD (Fig. 1). The modification consists on the addition of 6 mM EDTA, 0.05% digitonin, 0.01% sodium tripolyphosphate, and 100 μg/mL heparin to the reaction mixture. By using this procedure, we achieved detection of prions in dilutions higher than 100 million‐folds of the brain (10^−8^ dilution) (Fig. 1). For the experiments, we used as a substrate for PMCA human PrP^C^ containing either M or V at position 129. The results suggest that in a PMCA reaction the two substrates behave similarly except perhaps in MM1 cases, where the V isoform is less efficient for conversion. Next, we investigated whether urine interferes with the assay by spiking healthy urine with these brain dilutions. As shown in Figure 2, after 3 rounds of PMCA, we were able to observe a similar detection level between brain dilutions done either in buffer or urine. A significantly lower level of detection was achieved after 1 or 2 rounds of PMCA (Fig. 2), which likely reflects PMCA inhibition by either urinary compounds or by the presence of IOME beads used during urine processing.

**Figure 3:**
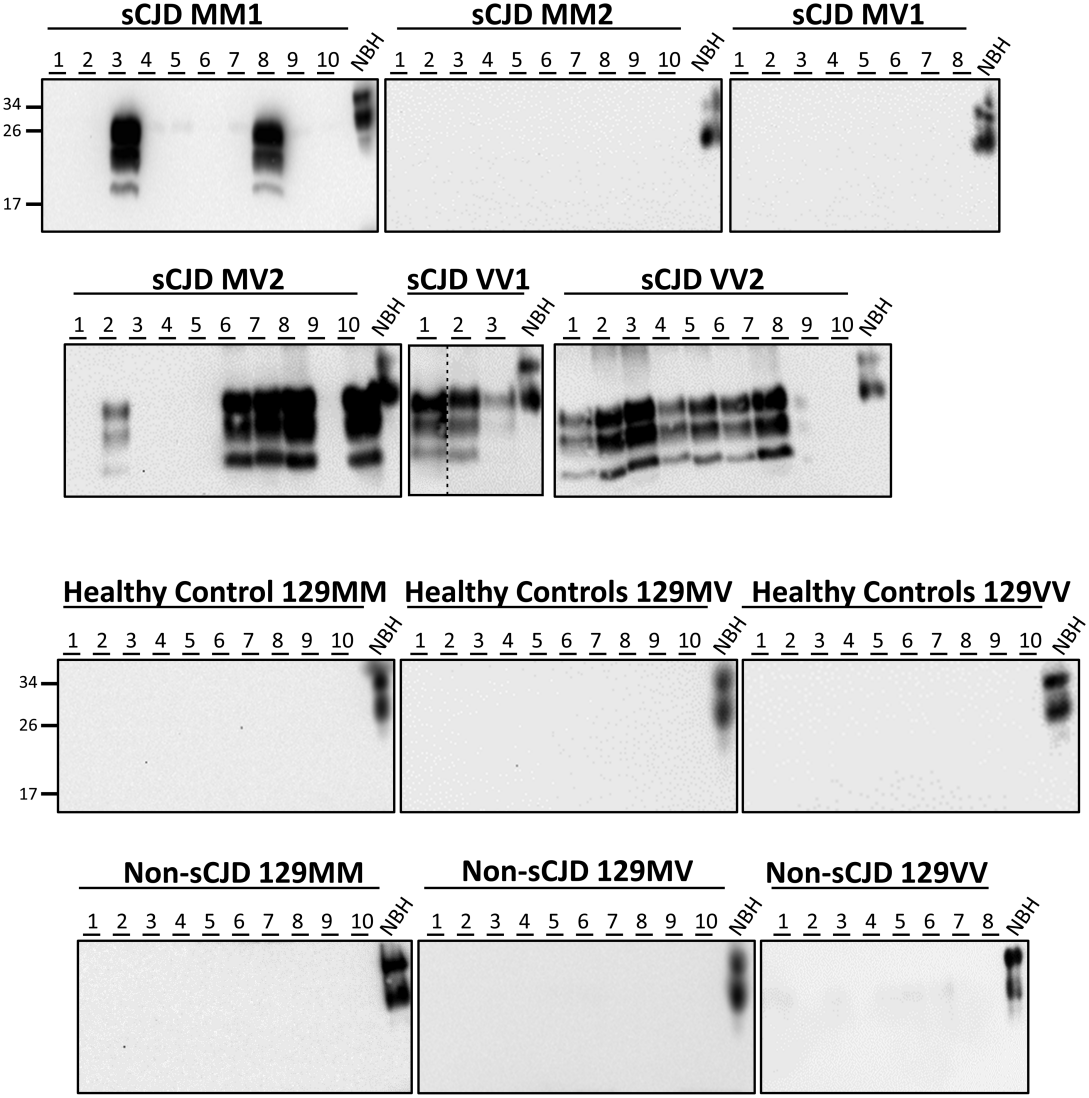
Representative detection of sCJD prions from patient urine samples. The figure shows the results of western blots from samples amplified by three rounds of PMCA starting with urine from different sCJD patients and controls (both healthy individuals and patients affected by other neurological diseases). Each number represents a urine sample from a different patient. For most sCJD subtypes, results from 10 samples are shown, except for MV1 and VV1 where we were able to obtain only eight and three patient samples, respectively. All MM2 samples analyzed in this study were MM2C. Prior to PMCA, urine samples were processed by IOME extraction as described in methods. To further evaluate the sensitivity of PMCA detection, urine samples were evaluated in both 129M and 129V substrates. The dotted line indicates blot splicing to remove nonrelevant lines.

When samples of urine from sCJD patients were tested, we observed a substantial number of positive samples, which varied depending on the subtype of sCJD (Fig. 3). Importantly, no signal was observed in any of the urine samples coming from healthy individuals or controls affected by other neurological or neurodegenerative diseases. We did not observe any effect of age in the healthy control groups; basically, all samples were negative regardless of the age. Thus, we pooled data from people at distinct ages. Overall, for sCJD samples, we detected PrP^Sc^ in 29 of the 81 sCJD cases studied (Table 1). The highest detection rate was observed in VV1 (100%), followed by VV2 (84%). Lower sensitivity was seen in MV2 and MM1, and no detection was seen in any samples of MV1 and MM2 (Table 1). The MM2 urine samples tested in this study were all coming from MM2C patients. To evaluate statistical differences between the groups, we compared whether each sCJD subtype produced a different result from the average of the entire group using the Fisher's exact test. This analysis showed that the MM2, MV1, VV1, and VV2 results were significantly different from the results obtained in the entire group of sCJD samples. This makes sense since VV1 and VV2 have the highest sensitivity and MM2 and MV1 have the lowest sensitivity. Considering the results of the test, the positive predictive value is 1.0 (0.883 to 1.0 95% confidence interval) and the negative predictive value of the test is 0.74 (0.68–0.80 95% confidence interval).

**Table 1:** Overview of urine samples and detection by PMCA.

Clinical diagnosis	PMCA positive/total samples analyzed
sCJD MM1	5/24
sCJD MV1	0/8
sCJD VV1	3/3
sCJD MM2	0/16
sCJD MV2	5/11
sCJD VV2	16/19
sCJD all types	29/81
Other neurological diseases	0/94
Healthy controls	0/66

## Discussion

This study demonstrates that PrP^Sc^ is detectable in urine of some sCJD cases depending on the disease subtype. This finding contrasts with our previous report that PrP^Sc^ was not detectable by PMCA in sCJD urine samples, but only on vCJD urine.^7^ However, the previous study was done with a version of PMCA that is not very efficient at detecting sCJD prions. In the present work, we modified the assay to increase its sensitivity to detect sCJD prions. Under the new conditions, prions from all major subtypes of sCJD can be detected up to quantities equivalent to 10^−7^ or 10^−8^ dilutions of diseased brains. Using the optimized assay, we detected PrP^Sc^ in urine of 36% of the sCJD patients examined and in none of the controls. The positive predictive value was 1.0 (100%), indicating that whenever the test detects a positive sample it is likely to be positive. Detection was sCJD subtype dependent with PrP^Sc^ containing VV at position 129 like in the VV1 and VV2 subtypes showing sensitivities of urinary prion detection of 100% and 84%, respectively. Combined, these two subtypes account for approximately 16–20% of all cases of sCJD.^11^ The reason for the distinct detectability of PrP^Sc^ in urine in different sCJD subtypes is unclear, but it may reflect different paths of PrP^Sc^ propagation affecting distinct areas of the brain and perhaps extracerebral tissues. Indeed, a recent study has shown that the first regions of brain damage and propagation paths are drastically different in the VV2 and MM1 subtypes.^19^ It is also possible that an underlying inflammation in some patients may have increased PrP^Sc^ secretion in urine, as has been shown in animal models by Aguzzi and coworkers.^20^

Our results are similar to those reported previously by Jackson, Collinge and coworkers using a methodology originally used for vCJD prion detection in blood involving the capture of disease‐associated PrP on a stainless steel matrix and detection using anti‐PrP monoclonal antibodies.^12^ The authors reported PrP^Sc^ detection in 8 of 20 sCJD urine samples analyzed (40% sensitivity). However, the low number of samples tested precludes a detailed comparison on detectability by sCJD subtype. Nevertheless, the similar level of sensitivity for PrP^Sc^ detection in urine obtained in both studies supports the concept that only a subset of sCJD patients excrete prions in urine.

Our findings suggest that PrP^Sc^ is detectable in urine samples, suggesting it might be a potential risk for transmission. However, it is important to highlight that PrP^Sc^ was detectable only after extensive PMCA amplification, suggesting that the concentration of infectious prions in urine is exceedingly small. Indeed, experimental studies have failed to transmit sCJD MM1 following urine inoculation to highly receptive transgenic mice even after 100X concentration and dialysis of the patient's urine.^21^ Furthermore, epidemiologic evidence suggests that family caregivers and medical personnel (especially nurses) are not at increased risk of developing sCJD compared to the general population.^22^ However, various medications, mostly for infertility, are still extracted from human or animal urine,^23^ representing a potential risk for iatrogenic prion transmission. Although a number of precautions are taken, the procedures used to purify and concentrate these products might co‐purify or even enrich PrP^Sc^ in these samples. Perhaps, testing for the presence of prions by PMCA or other methodologies on the final product should be considered. Finally, prion detection in urine might be used as a noninvasive preliminary screening test to detect sCJD. Future effort should enable to improve the sensitivity of the test by either increasing PMCA efficiency or concentrating the sample. Another alternative we are exploring is to use plasma, since most of the material secreted in urine also circulates in plasma in larger concentrations.

## Author Contributions

SP participated in the design of the experiments, supervision of the work, analysis of the data, preparation of figures and wrote the first draft of the article. FR and AL performed the PMCA experiments. PS, FM, BA, SN, and PG provided samples for the studies. SR performed the statistical analysis. CS supervised the entire project, acquired funding, and was responsible for the final version of the manuscript. All authors reviewed and corrected the manuscript.

## Conflict of Interest

Claudio Soto is a Founder, Chief Scientific Officer and Member of the Board of Directors of Amprion Inc, a biotechnology company that focuses on the commercial use of PMCA and other seed amplification assays for high‐sensitivity detection of misfolded protein aggregates involved in various neurodegenerative diseases. Sandra Pritzkow also has a conflict in relation to Amprion. The University of Texas Health Science Center at Houston has licensed patents and patent applications to Amprion.

## Data Availability

The original contributions presented in the study are included in the article/Supplementary Material; further inquiries can be directed to the corresponding author.
